# Hospital Meetings

**Published:** 1903-02-07

**Authors:** 


### HOSPITAL MEETINGS.

### THE ITALIAN HOSPITAL: ITS CLAIMS ON THE BRITISH POPULATION.

The Italian element, as was to be expected, predominated on Saturday afternoon (31st ult.) when the annual general meeting of the above hospital took place. With two exceptions, all those present were members of the committee of management. They were Mrs. Angiola Ortelli, Miss Bianconi, Cav. Luciano de Rin (Hon. Sec.), Lord Edmund Talbot, M.P., General Ramsay Slade, C.B., Cav. Dr. F. Naumann, Dr. Vincent Dickinson, Cav. Dr. M. Castaneda, Mr. F. Miller, Cav. P. Righette (Vice-Consul), Mr. Federico Benito, Cav. Micali, and the Very Rev. J. P. Baunin, Hon. Chaplain.

The chair was taken by His Excellency the Ambassador Extraordinary and Minister Plenipotentiary of his Majesty the King of Italy. He made no remarks on the report, which was printed both in Italian and English, and taken as read. Cav. Luciano de Rin, in seconding its adoption, said he wished to point out that the number of the patients had increased by 80, and the out-patients by 2,008, during last year. The balance-sheet showed a balance in hand of £1,099 ; subscribers however must not run away with the idea that this was saved from last year ; it included £600 from King Edward's Hospital Fund, of which £500 was an annual subscription, and £100 a special donation. This was received in December, and although it appeared in the balance-sheet, it was to be devoted to meeting the expenditure in the current year.

In seconding the re-election of the Committee of Management, General Slade remarked that, large as was the number of Italians treated as out-patients at that hospital, the number of British was still larger. Since the hospital was founded, up to the end of last year, 31,239 Italians and 34,132 British out-patients had been treated. Moreover, 725 British in-patients had been admitted. These facts, he thought, called for support from all classes, not only of Italians, but also of British nationality, resident in London. With regard to the admission of patients, there was no restriction beyond the fact that if two persons applied, one being Italian and the other British, preference was naturally given to the former. Economical management had been maintained during the past few years, and the committee had done their best to maintain the hospital on a sound financial basis.

Closed Beds Re-opened.

The resolution next on the agenda was as follows : "That the sincere and dutiful thanks of the governors be, and are hereby tendered with the greatest respect to H.R.H. the President of King Edward's Hospital Fund for London, for the yearly grant of £500 awarded from the Fund (to enable the 10 remaining beds to be opened), and the donation of £100 which his Royal Highness has graciously made to the hospital."

The President, in moving it, said the resolution spoke for itself. Beds that had been closed for want of funds were now, thanks to King Edward's Hospital Fund, re-opened. Although a brighter prospect was thus opened up for the coming year, the same help from subscribers would be required as in former years, since the re-opening of the 10 beds would increase the expenditure. He therefore appealed to all who were in sympathy with an institution that helped all in need, regardless of nationality.

Mr. Francis Miller emphasised the fact that the hospital helped not only the Italians in that quarter, but also to a very large extent the English. He recognised in the donation of King Edward's Fund a certain admission that the hospital, after being inquired into by the visitors, had been found worthy of their support, and he expressed the gratitude of the committee for their "handsome donation." "We can still," he added, "fill as many beds as we have, and the only anxiety we have to look forward to is that of funds to enable us to keep up the hospital in the state in which it ought to be." He thought the English should come forward a little more than they had done, to help forward this good work.

### Hospitals and the Press.

Lord Edmund Talbot proposed a vote of thanks to the editors of the British and Foreign Press for their courtesy and much valued help, freely and repeatedly extended to the hospital throughout the past year.

### The President.

Cav. Dr. M. Castaneda, in proposing a vote of thanks to the president, said there were four ways in which His Excellency had helped in a special manner. He presided at the annual meeting, always a red-letter day; he helped to make the annual ball a success; he organised the visit of the Duke and Duchess of Aosta in June, on which occasion Madame Pausa also accompanied the party; and finally, he was instrumental in obtaining the magnificent grant from King Edward's Fund. The ball, instituted at Mrs. Ortelli's suggestion, was last year so successful as to bring in £800.

Lord Edmund Talbot said he looked upon the supplementary donation from King Edward's Fund as an acknowledgment of the good management of the hospital, on which their best hopes for the future were based.

